# Effects of Rhizome Integration on the Water Physiology of *Phyllostachys edulis* Clones under Heterogeneous Water Stress

**DOI:** 10.3390/plants9030373

**Published:** 2020-03-18

**Authors:** Xiong Jing, Chunju Cai, Shaohui Fan, Guanglu Liu, Changming Wu, Benxue Chen

**Affiliations:** International Center for Bamboo and Rattan, State Forestry and Grassland Administration Key Laboratory of Bamboo and Rattan, Beijing 100102, China; jingx@icbr.ac.cn (X.J.); liuguanglu@icbr.ac.cn (G.L.); 15761635165@163.com (C.W.); benxuechen@126.com (B.C.)

**Keywords:** Heterogeneous water stress, *Phyllostachys edulis*, Rhizome, Vascular bundle, Stress Signal, Physiological characteristics

## Abstract

Water is crucial to plant growth and development. Under heterogeneous environmental water deficiency, physiological integration of the rhizomatous clonal plant triggers a series of physiological cascades, which induces both signaling and physiological responses. It is known that the rhizome of *Phyllostachys edulis*, which connects associated clonal ramets, has important significance in this physiological integration. This significance is attributed to the sharing of water and nutrients in the vascular bundle of clonal ramets under heterogeneous water conditions. However, the physiological characteristics of physiological integration under heterogeneous water stress remain unclear. To investigate these physiological characteristics, particularly second messenger Ca^2+^ signaling characteristics, long-distance hormone signaling molecules, antioxidant enzyme activity, osmotic adjustment substance, and nitrogen metabolism, ramets with a connected (where integration was allowed to take place) and severed rhizome (with no integration) were compared in this study. The vascular bundle structure of the rhizome was also observed using laser confocal microscopy. Overall, the results suggest that interconnected rhizome of *P. edulis* can enhance its physiological function in response to drought-induced stress under heterogeneous water deficiency. These measured changes in physiological indices serve to improve the clonal ramets’ drought adaptivity through the interconnected rhizome.

## 1. Introduction

Recently, drought problems caused by global climate change have been on the rise. Drought is a common and severe problem in agriculture—in fact, damage caused by drought causes a much larger loss of livelihood and yield than other natural disasters [1,2]. Drought is a multidimensional stress and can significantly constrain plant productivity and trigger a wide variety of responses at a level concerning physiological, biochemical, and molecular aspects [3,4]. 

In China, the bamboo resources and the total area of bamboo forest rank among the largest in the world. There are more than 500 species of bamboo comprising 39 genera [5,6]. Bamboo is also one of the most important clonal plant resources [7]. Clonal plants are those in which vegetative growth is accompanied by cloned ramets, which can self-replicate and spread quickly under natural conditions by asexual reproduction [8,9].

In nature, water and nutrients needed for plant growth, are usually heterogeneously distributed, even at a very small scale [10]. In heterogeneous small-scale environments, physiological integration and foraging behavior are important characteristics of clonal plants, among which physiological integration is the internal driver of clonal growth in heterogeneous habitats. In these plants substances can be transferred via the vascular system of the clonal ramets [11,12]. Clonal plants depend on an extensive rhizome-root system, which has the ability to extend through a large area and create many clones. The rhizome acts as a spacer and is an important vascular system for physiological integration of clonal plants [13,14]. The rhizome of moso bamboo (*Phyllostachys edulis*) acts as a spacer for resource sharing between cloned ramets of *P. edulis* [15]. In a heterogeneously distributed resource environment, the *P. edulis* rhizome system plays an important role in transporting water, photo-assimilates, and carbohydrates under a source-sink gradient [16,17,18].

Clonal growth and physiological integrations in clonal plants have been an emerging field of research in recent years [11]. Existing research on clonal plants mainly focuses on dwarf herbaceous clonal plants such as *Zoysia japonica* and *Buchloe dactyloides* [19,20] It is worth noting that studies have found that amphibious clonal plants with a high capacity for clonal integration are useful for re-vegetation of degraded aquatic habitats caused by Cd contamination [8]. This provides precedent for the use of clonal plants to reduce heavy metal pollution in other habitats. However, research into the physiological integration of bamboo is still in the preliminary stage, and related research exists mainly focuses on describing the phenomenon and on the intensity of integration. 

Under heterogeneous water stress, the relationship between rhizome length and physiological integration of *Indocalamus decoru* has been studied. It was found that as the rhizome length increased, the water potential gradient decreased, that is to say, the integration of water between the donor ramet and the recipient ramet decreased [21]. Clonal integration (resource translocation) between connected ramets of clonal plants can increase their tolerance to stress. However, there are few studies on the physiological characteristics of water translocation in clonal integration. We postulate that both the signaling and physiological responses play a significant role in modulating the physiological integration of *P. edulis*. 

To fill this research gap, an experiment was conducted under heterogeneous water content of the soil (RWC = 25 ± 5%, 80 ± 5%) on *P. edulis* with both connected and disconnected rhizomes. Concentrations of the long-distance signaling molecules abscisic acid (ABA) and jasmonic acid (JA) were monitored. Instant flux of the second messenger Ca^2+^ in pilorhiza, rhizomes, and leaves was considered the major signaling procedure. In addition, physiological responses such as endogenous hormone concentration, antioxidant enzyme activity, osmotic adjustment substances, NO^3−^, and NH^4+^ flux in different water patches in the clonal ramets were investigated, and rhizome and stem structure were observed under laser confocal microscopy (CLSM) to gain a better understanding of the vascular bundle function. 

## 2. Results and Discussion

### 2.1. Morphological Basis of P. edulis Physiology Integration and Analysis of Its Influencing Factor

*P. edulis* is a monocotyledonous plant, and the vascular bundle does not contain cambium. The vascular bundle is instead composed of primary phloem and primary xylem, wherein the primary xylem includes vessel or tracheid cells, parenchyma, and fiber. The primary phloem includes sieve elements, sieve cells, and companion cells. Lignin deposition is an important step in the formation of the vascular bundle, so we took advantage of the high lignin content in the vascular bundle and recorded lignin autofluorescence using laser confocal microscopy.

**Figure 1:**
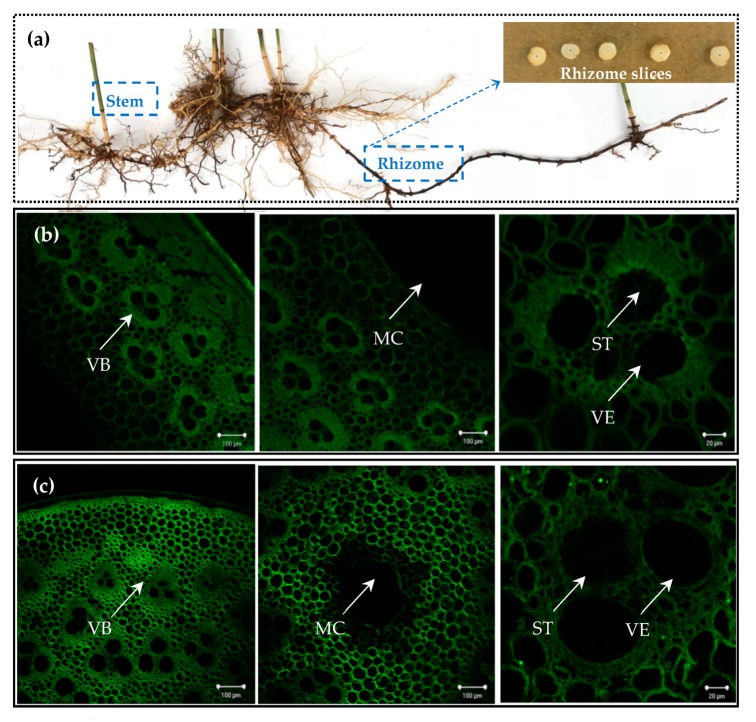
The Laser Confocal Scanning Microscop (LCSM) images of the *P. edulis* stem and rhizome structure. (**a**) The display of *P. edulis* sprouting seedling test material; (**b**) represents the structure of the stem slices; (**c**) represents the structure of the rhizome slices. The magnification is set a ×10, ×10, and ×40 times (left to right) the original size. Vascular bundles were denoted as (VB), medullary cavities were denoted as (MC), sieve tubes were denoted as (ST), and vessel elements were denoted as (VE).

As shown in Figure 1, the structure of both the rhizome and the stem vascular bundles was observed by lignin autofluorescence using a laser confocal microscope. Both the structure in the rhizome vascular bundle (Figure 1c VB) and that of the stem (Figure 1b VB) were composed of two vessel elements (VE) and a sieve tube (ST) containing sieve holes. The vascular bundles are arranged in a V-shaped scattered arrangement on the transverse section. The bottom of the V-shape is a sieve tube (ST) and the arms of the V-shape each have two vessels (VE). The bottom of the V-shape vascular bundles on the rhizome and stem respectively are oriented towards their own cortex, meanwhile the V-shaped arms are each oriented towards their respective medullary canal.

However, in the stem, a medullary ring and a large medullary cavity was found whilst in the rhizome, there was no medullary ring and only a small medullary cavity. From the perspective of the xylem developmental structure, the primary xylem of both the rhizome and stem belonged to the endarch type (referring to proto xylem being directed towards the inner center). In botany, the underground spacing mechanism of the *P. edulis* plant is the rhizome, which is a metamorphosis of the stem, so in effect, the rhizome and stem share the same vascular bundle structure. The vascular system provides objective conditions for the physiological integration of cloned ramets. The interconnected vascular system of *P. edulis* facilitates the transfer and sharing of substances under heterogeneous resource distribution, such as water, nutrients, and photosynthetic products, which are carried along the gradient of material in source-sink relationships. Therefore, physiological integration of the cloned ramets of the *P. edulis plant* is achieved by the rhizomes connected to each other. The rhizome is used as the organ for material transfer between the cloned ramets, and the shared distribution of resources in the heterogeneous plaque environment plays an important role. 

The Munch pressure flow theory stipulates that the transport flux of assimilates in the vascular bundle is driven by the swell gradient, which can be understood by means of the flux calculation formula (J = δP·C·k, where δP is the gradient of turgor between source and sink, C is the concentration of assimilates, and k is equal to the conductivity coefficient) of the assimilates in the vascular bundle [22]. If NC (clonal ramet in normal water treatment) is considered to be a metabolic source, then water and organic matter can be transferred to the metabolic sink (SC, water deficiency of clonal ramet). The transport capacity of the phloem is mainly affected by the cross-sectional area of the sieve tubes and the distance of the source-sink. How regularly the rhizome grows is also related to the source- sink relationship [23]. In a heterogeneous water environment, the vascular bundle in the rhizome plays an important role in the resource and material distribution of the *P. edulis* plant, hence the age and length of the rhizome are important factors.

### 2.2. Effects of Rhizome Integration on the Related Physiological Indexes of P. edulis Clones under Heterogeneous Water Stress

Under the influence of varying soil relative water content (RWC = 25 ± 5%, 80 ± 5%), rhizome status, a variance analysis of the leaf related physiological indexes of three-year-old *P. edulis* sprouting seedlings is shown in Table 1. A model was designed for the analysis of variance of nine physiological indicators such as leaf enzyme activity (SOD, CAT, and POD), hormone content (JA, ABA, and IAA) and osmotic adjustment substance (MDA) content, etc. The model is: Y=AX_1_+BX_2_+CX_1_X_2_+D+e, among which X_1_, X_2_ represents water and rhizome status respectively; D indicates the intercept; e is the error; and ABC is the coefficient (Table 1). 

It can be seen from Table 1 that both water stress and a combination of water and rhizome status factors had a significant effect on each relevant physiological index (*p* < 0.001). However, rhizome status had a significant impact on all indicators except for SOD, POD, and Pro. This means, the effect of water on the physiological function of the cloned ramets of *P. edulis* was affected by the status of the rhizome. The influence of the rhizome status on the physiology of the plant was also closely related to the existence of heterogeneous water treatment. It can be concluded that the rhizome plays an important role in heterogeneous drought stress physiology.

**Figure 2:**
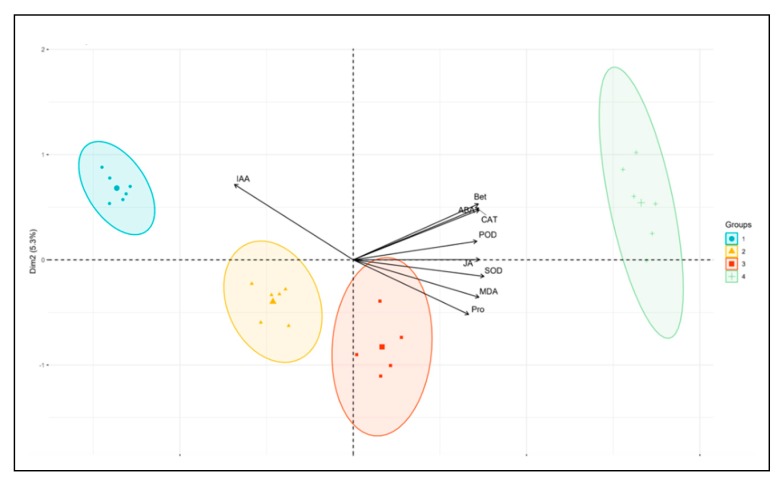
PCA analysis between different physiological indicators of *P. edulis* sprouting seedlings. ND treatments were denoted as 1 Groups, NC treatments were denoted as 2 Groups, SC treatments were denoted as 3 Groups, and SD treatments were denoted as 4 Groups.

Principal component analysis (PCA) was used to analyze the correlation between the nine physiological factors including leaf enzyme activity (SOD, CAT and POD), hormone content (JA, ABA and IAA) and osmotic adjustment substance (MDA) content (Figure 2). The results show that the contribution rate of the variance of the first principal component is 88.5%, the second principal component variance contribution rate was 5.3%, making a total contribution of 93.8%. The first and second principal components could reflect the differences of the nine physiological indexes among different groups. There is a significant correlation among various physiological indicators, IAA was significantly negatively correlated with other physiological indicators, and all physiological indicators except IAA were significantly positively correlated. In addition, each physiological index is clearly divided into four groups (water and rhizome status) on the principal component axis, and the physiological indexes between different groups are significantly different, that is, water and rhizome status have a significant impact on physiological indexes of *P. edulis*.

### 2.3. Effect of the Rhizome on the Ca^2+^ Flux as the Second Messenger of P. edulis under Heterogeneous Water Stress

**Figure 3:**
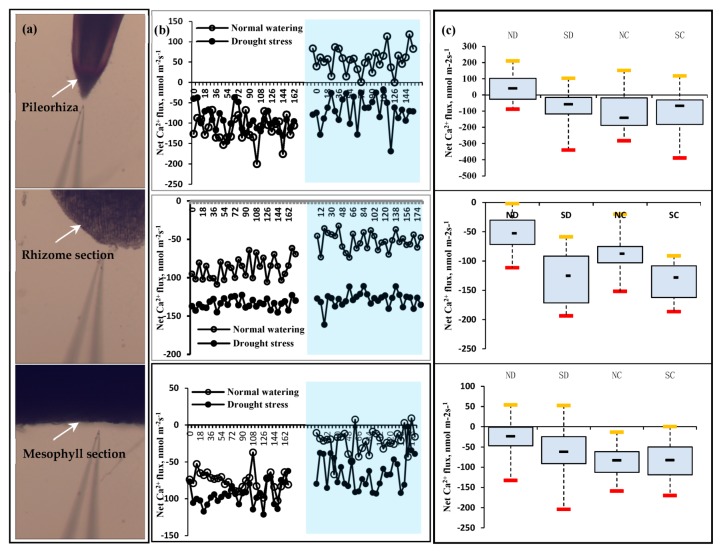
Effect of the rhizome on Ca^2+^ flux in different organs of clonal ramets under heterogeneous water stress. (**a**) Schematic of Ca^2+^ fluxes test in the pileorhiza, rhizome transection, and leaf transection of *P. edulis* seedling, respectively; (**b**) Ca^2+^ flux dynamic in clonal remats of *P. edulis* sprouting seedlings under heterogeneous water stress. The white area represents the Ca^2+^ oscillation in the connected rhizome treatment of the *P. edulis* seedlings, and the blue region indicates Ca^2+^ oscillation under disconnected rhizome treatment; (**c**) Box plot shows Ca^2+^ flux range in the different organs tested.

NMT was used to monitor Ca^2+^ flux in the pileorhiza, mesophyll transverse section, and rhizome transverse section in vivo, the flux rule is shown in Figure 3. Analysis of significant difference showed that Ca^2+^ fluxes in cloned ramets ND (treatment of disconnected clonal ramet in normal water) and SD (treatment of disconnected clonal ramet in water stress) were significantly different in pileorhiza, in the rhizome transverse section and in the mesophyll transverse section (*p* < 0.05), which showed that the pileorhiza of the SD had stronger Ca^2+^ absorption capacity than the ND. However, there was no significant difference in Ca^2+^ flux between the pileorhiza and mesophyll transverse section of the NC (treatment of connected clonal ramet in normal water) and SC (treatment of connected clonal ramet in water stress) cloned ramets in the connected rhizome treatment under heterogeneous water. 

The rhizome of connected clonal ramets shared water, which is assumed to be due to the source-sink gradient between cloned ramets [21]. There was a smaller difference in Ca^2+^ signal intensity in difference organs of the cloned ramets in the connected rhizome treatment, which might be related to the water “equalization” effect of inter-clonal ramets on the heterogeneous drought stress. This is that the water or substance of the sink-clonal ramet SC was transported by the ramet NC. The difference in the Ca^2+^ flux between the leaves of the NC and SC treated plants and the associated transverse section of the rhizome was not obvious in this shared transport mechanism. 

It is understood that ABA can induce the intracellular production of reactive oxygen species (ROS) such as active hydrogen peroxide (H_2_O_2_) under drought stress. ROS in turn act as post-stress messengers, activating Ca^2+^ channels in the plasma membrane to release Ca^2+^ from the vacuole to the cytosol. The influx of extracellular Ca^2+^ can also initiate intracellular Ca^2+^ oscillations and promote the release of Ca^2+^ from the vacuole to the cytosol [24].

However, due to the complexity of Ca^2+^ flux signaling in the rhizome of *P. edulis*, related research is limited at present. It is possible that there the lack of difference in the Ca^2+^ signal flux in the cloned ramets NC and SC under heterogeneous water stress was due to other factors. Studies have shown that Ca^2+^ signaling can regulate plant physiology to improve the physiological response to drought stress; Ca^2+^ is also related to long-distance signaling substances such as ABA [25,26]. A study conducted showed that the differentiation of ductal molecules required the initiative of a Ca^2+^ influx pathway [27]. Meanwhile, the concentration of free Ca^2+^ in the sieve-tube was nearly 20–100 times higher than that in general cells. It was speculated that the concentration of Ca^2+^ is related to the transport direction of organic matter [28]. In research conducted on bamboo, Yu found that the phloem ganglion showed extracellular Ca^2+^ transfer into the cell as the phloem ganglion developed, and Ca^2+^ regulated the physiological function of the phloem ganglion [29]. 

The box plot shows that Ca^2+^ oscillation in the rhizome of the cloned ramets NC and SC was smaller than that of ND and SD. By comparing the four treatments, it is apparent that the oscillation amplitude of Ca^2+^ flux in the NC cloned ramet was the smallest of the four treatments and that the overall order of Ca^2+^ oscillation amplitude was NC < ND < SC < SD. However, this rule does not apply to the other plant organs tested, such as the pileorhiza and mesophyll layers (Figure 3c).

### 2.4. Effect of Rhizome Integration on Endogenous Hormone Concentration in the Leaves of P. edulis under Heterogeneous Water Stress 

**Figure 4:**
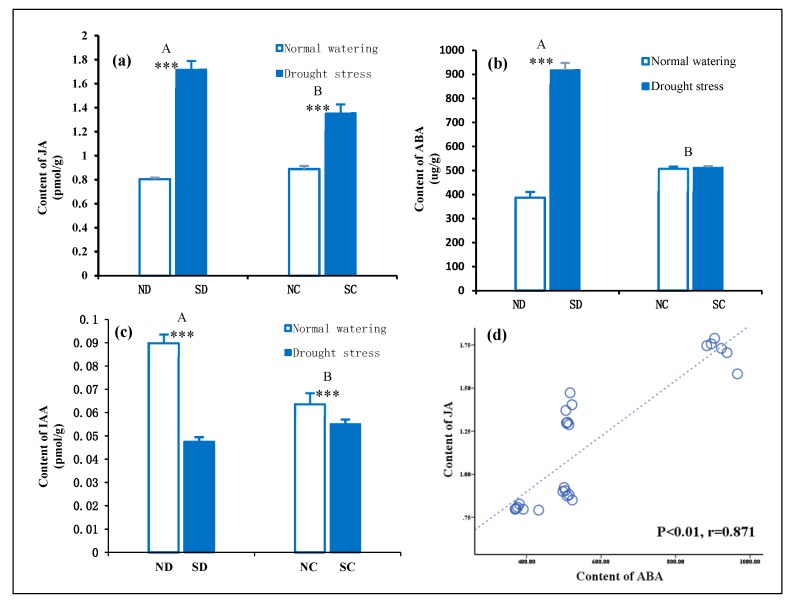
Effect of the rhizome on the changes of endogenous hormones in leaves of clonal ramets under heterogeneous water stress conditions. Letters (**a**–**c**) indicate JA, ABA, and IAA concentrations in the leaves of the four treatments respectively. Bars are mean values (±SE, n = 4). Different capital letters in the figure indicate significant differences between connected and disconnected (*p* < 0.01). Symbols indicate levels of statistical significance between normal watering (RWC = 80 ± 5%) and drought stress (RWC = 25 ± 5%) treatments for rhizome treatment: no symbol *p* > 0.1; *** *p* < 0.001. (**d**) Statistical correlation analysis between JA and ABA concentration in the leaves (y = 0.0016x + 0.2549, R^2^ = 0.759; *p* < 0.01; Pearson correlation coefficient, r = 0.871.).

Plant hormones, such as JA and ABA, play an important regulatory role under drought stress, and also act as important signal regulators. Figure 4 shows the concentration of JA, ABA, and IAA in the leaves of *P. edulis* with rhizomes treatment by comparing severed (preventing integration) and connected rhizomes (allowing integration) under heterogeneous water stress. The concentration of JA and ABA in the leaves of the cloned ramets were significantly correlated (*p* < 0.01, r = 0.871), indicating that the endogenous hormones JA and ABA act as adversity sensors and initiate the stress metabolism under heterogeneous drought stress [30]. Moreover, ABA and JA may have synergistic effects in response to drought stress. ABA and JA may play a key role in perceiving the heterogeneous water environment between two clonal ramets. Interestingly, changes in ABA and JA concentration in the leaves of the connected cloned ramets were different; there was no significant difference in ABA concentration, but there was a significant difference in JA concentration.

It can be seen from Figure 4 that under heterogeneous water stress, abscisic acid (ABA) and JA concentration in the *P. edulis* plant with a connected rhizome were significantly lower than that of the disconnected rhizome. After the plant was subjected to drought stress, ABA was found to act as a long-distance signal produced in the pileorhiza, which was transmitted along with water to the aerial part through the xylem vessel to regulate the stomatal opening of the plant leaves under drought stress. This mechanism reduced transpiration [31,32]. 

JA is transported from the top of the plant to the bottom of the plant by the phloem. Studies have shown that JA is produced in the plant leaves, then transported from the leaves by the phloem (screen) to the roots. JA transfer accelerated under water stress and the upward transfer of ABA was in turn promoted [33,34,35,36]. This indicates that the response sites of ABA and JA under drought stress are different, which may be one of the reasons that the difference in JA concentration between NC and SC leaves of cloned plants is significant, but the difference in ABA concentration is not significant.

Polar transport of IAA is observed in the plant from the top to the bottom of the stalk, and the plant can produce a variety of physiological effects as the concentration of IAA changes [37]. Under heterogeneous water stress, the IAA concentration of the clonal SC was significantly lower than that of the NC. However, when compared with the difference between ND and SD leaves, the difference in IAA concentration between cloned NC and SC leaves was much lower (3.9 times lower) (Figure 4). The effect of the rhizome treatment (connected rhizome and disconnected rhizome) on the endogenous hormone concentration in a heterogeneous water environment is considered to be the result of the rhizome vascular system participating in the sharing of water and material distribution. From the perspective of the source-sink relationship, the NC-treated clonal ramets supplied water to the drought stress treatment clonal SC, which not only relieved the stress of the clonal strain SC, but also improved the supply equilibrium fitness of the whole plant to the heterogeneous stress environment. 

### 2.5. Effect of Rhizome Severance on the Antioxidant Enzyme System in the Leaves of P. edulis under Heterogeneous Water Stress 

Abscisic acid (ABA) was induced by biological and abiotic stress stimulation and the production and accumulation of reactive oxygen species (ROS) [38]. When the plant was subjected to drought stress, a large amount of ROS, such as superoxide anion radicals (·O^2−^), hydrogen peroxide (H_2_O_2_), and hydroxyl radicals (·OH) were produced in vivo. ROS may have toxic effects on the growth and development of plants. The plant removes ROS through the antioxidant enzyme system. In the cellular antioxidant defense system, SOD, CAT, and POD are all regulated by ROS, and their reaction effects can be synergistic [39]. Excess ·O^2−^ produced in the plant cells is disproportionated into H_2_O_2_ and O_2_ under the catalysis of SOD, and H_2_O_2_ is further dismutated into O_2_ and H_2_O under the catalysis of hydrogen peroxide dismutase CAT and peroxidase POD. Through the interaction of these three enzymes, the plant can effectively control ROS accumulation and ensure the continuation of normal physiological functions under drought stress [40]. 

**Figure 5:**
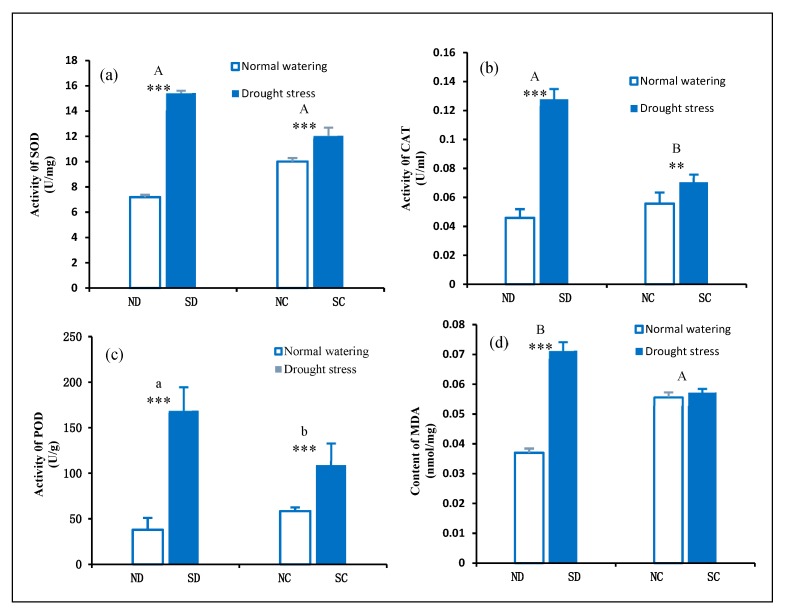
Effects of the rhizome on the changes of antioxidant enzyme activities and malondialdehyde concentration in leaves of clonal ramets under heterogeneous water stress. (**a**–**c**) represent SOD, CAT, POD activity of *P. edulis* leaves among the four treatment, respectively. (**d**) MDA concentration of *P. edulis* leaves under different treatment. Bars are mean values (±SE, n = 4). Different capital letters in the figure indicate significant differences between connected and disconnected (*p* < 0.01). Different lowercase letters indicate statistical significance differences (*p* = 0.01–0.05). Symbols indicate levels of statistical significance between normal watering (RWC = 80 ± 5%) and drought stress (RWC = 25 ± 5%) treatments for rhizome treatment: no symbol *p* > 0.1; ** *p* = 0.001–0.01; *** *p* < 0.001.

As shown in Figure 5, SOD, CAT, and POD activity in the leaves of ND and SD were significantly different (*P* < 0.001). The leaves of ND showed low activity of these substances, and the SD showed high activity. When only taking the connected/disconnected rhizome factor into account, it was found that CAT and POD activity was considerably weaker in the connected rhizome treatment than in the disconnected treatment. SOD activity was not significantly different across the two treatments. This may be due to the fact that the clonal ramets of the *P. edulis* plant are able to perceptively sense heterogeneous environmental stress and affect physiological metabolism through the rhizome itself. The presence of the rhizome significantly affects the antioxidant enzyme metabolism of the source- sink clonal ramets, and improves the anti-ROS ability of the NC cloned ramets. 

One of the inevitable consequences of drought stress is an increase in reactive oxygen species (ROS) production in different cellular compartments [4]. When the concentration of intracellular ROS increases, an increase in antioxidant enzyme activity and plant antioxidant capacity is induced. However, when the plant is under environmental stress for a long time, ROS production and scavenging by the antioxidant enzyme system become unbalanced, which causes the accumulation of MDA in the plant [41]. The results of this study showed that under heterogeneous drought stress, the MDA concentrations in the connected rhizome between clone ramets NC and SC were not significantly different. However, there were significant differences in the MDA concentration between the disconnected rhizome cloned ramets ND and SD (Figure 5d).

The results indicated that under the same degree of heterogeneous drought stress treatment, the structural function of the connected rhizome may be the cause of these physiological effects. On the other hand, under heterogeneous water stress conditions, the rhizome does have an effect on ROS accumulation in cloned plants. The rhizome was an important hub for the source-sink relationship of *P. edulis*. 

### 2.6. Effects of the Rhizome on Osmotic Adjustment Substances in P. edulis Leaves under Heterogeneous Water Stress 

Osmotic adjustment is an important physiological regulation mechanism for plants to adapt to drought stress [42]. ROS produced in the leaves under drought stress can lead to increased membrane lipid permeability and electrolyte extravasation. By accumulating osmotic adjustment substances such as Pro and Bet, the cell’s osmotic potential can be reduced and cell swell pressure maintained [2].

Under heterogeneous drought stress, the difference of Pro concentration between the ND and SD leaves of the disconnected rhizome by severing was significant (*P* < 0.001), while the clonal ramets NC and SC, with a connected rhizome, have a difference in the leaves of Pro concentration which was found to be significant at *P* < 0.05. At the same time, Bet concentration under the disconnected rhizome treatment was extremely significant, but there was no significant difference in the Bet concentration under the connected rhizome treatment. 

**Figure 6:**
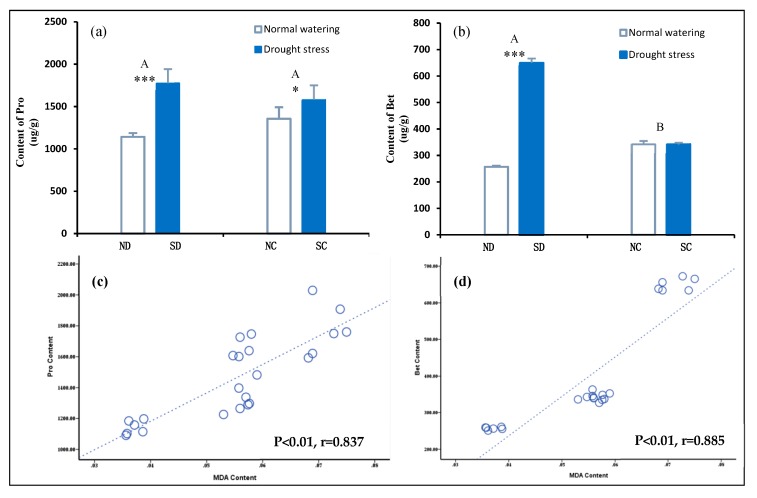
Effects of the rhizome on the changes of the concentration of substance in of clonal ramets under heterogeneous water stress. (**a,b**) represent Pro, Bet concentration in *P. edulis* leaves among the four treatment, respectively. Bars are mean values (±SE, n = 4). Different capital letters in the figure indicate significant differences between connected and disconnected (*p* < 0.01). Symbols indicate levels of statistical significance between normal watering (RWC = 80 ± 5%) and drought stress (RWC = 25 ± 5%) treatments for rhizome treatment: no symbol *p* > 0.1; * *p* = 0.01–0.05; *** *p* < 0.001. In (**c**) dashed (y = 18406.8x + 445.9, R^2^ = 0.687) and (**d**) dashed (y = 10770.8x – 194.0, R ^2^= 0.783) lines represent the linear regression for Pro concentration and MDA concentration (r = 0.837, *p* < 0.01), Bet concentration and MDA concentration (r = 0.885, *p* < 0.01), respectively. When *p* < 0.05, Pearson correlation coefficient (r) indicates the correlation between Pro or Bet concentration and MDA concentration.

The difference of osmotic adjustment substances (Pro and Bet) between the NC and SC leaves of the clonal ramets is smaller than that of ND and SD ramets. This difference in the concentration of osmotic adjustment substances caused by the rhizome may be related to the physiological response of the rhizome in a heterogeneous water environment. Studies have shown that drought stress can induce the increase of Pro and ABA concentration in plants, and that Pro accumulation depends on ABA, while ABA accumulation is independent of Pro [43]. Under heterogeneous drought stress, ABA concentration in SD treated leaves was significantly higher than that in ND, but there was no significant difference in ABA concentration between NC and SC treated leaves (Figure 6b). According to Figure 6, this is similar to the change of Pro and Bet concentration in NC and SC treated leaves under the same treatment. Increase in intracellular Pro- and Bet- concentration, which act as a driver of permeability in vivo, can maintain a high level of osmotic potential in the cell. Their accumulation can in turn stabilize the structure and function of biomacromolecules.

The correlation between proline (Pro) and malondialdehyde concentration and betaine and malondialdehyde (MDA) concentration was analyzed (Figure 6c,d). It was found concentrations of osmotic adjustment substances Pro and Bet were strongly correlated with MDA concentration. Furthermore, it indicated that under heterogeneous water stress, the interaction of water availability and rhizome treatment affected the osmotic adjustment substances of each cloned ramet. The rhizome can thus be said to improve the adaptability of the clonal ramets to heterogeneous drought stress. 

### 2.7. Effects of the Rhizome on the Nitrogen Metabolism in the Leaves of P. edulis under Heterogeneous Water Stress 

NH^4+^ and NO^3−^ absorbed by the roots of the plant are transported to the aboveground organs through the vascular tissue, and the absorption of nitrogen can then be used for the synthesis of substances in various plant organs. Nitrogen metabolism in leaves is closely related to chlorophyll, protein, free amino acid concentration, and photosynthetic nitrogen-use efficiency (PNUE) [44]. 

**Figure 7:**
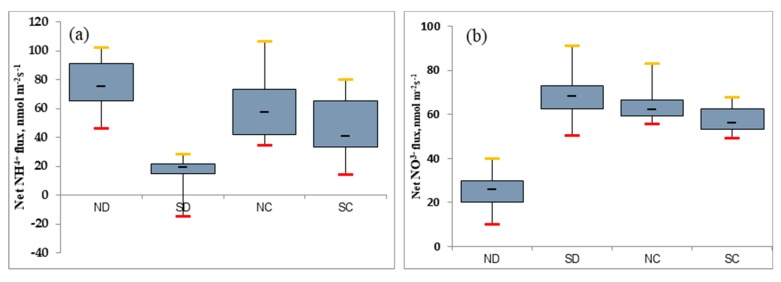
Nitrogen metabolism in leaves with either a connected or disconnected (severed) rhizome under heterogeneous water stress. (**a,b**) box plot shows NH^4+^ and NO^3−^ flux respectively.

Figure 7 indicates that under different treatments, the absorption of NH^4+^ and NO^3−^ by leaves showed an antagonistic trend. Compared with other treatments, the NH^4+^ efflux of SD leaves was the smallest, that is, the metabolic utilization of NH^4+^ in SD leaves under drought stress was increased, and the NO^3−^ metabolism absorption trend was opposite to that of NH^4+^ under the same treatment. From the NH^4+^ and NO^3−^ absorption flux, it was found that the NH^4+^ and NO^3−^ fluxes in the SC and NC leaves were not significantly different, while the difference in NH^4+^ and NO^3−^ between the SD and ND leaves in which the rhizome had been severed was larger.

Nitrogen metabolism in leaves has an important role in photosynthesis and protein synthesis. Under different treatments, the changes of NH^4+^ and NO^3−^ in leaves may be related to the absorption and metabolism of nitrogen in leaves. Combined with the above analysis, this shows that rhizome structure and water stress can both affect the nitrogen metabolism of *P. edulis* leaves.

## 3. Materials and Methods 

### 3.1. Materials

**Figure 8:**
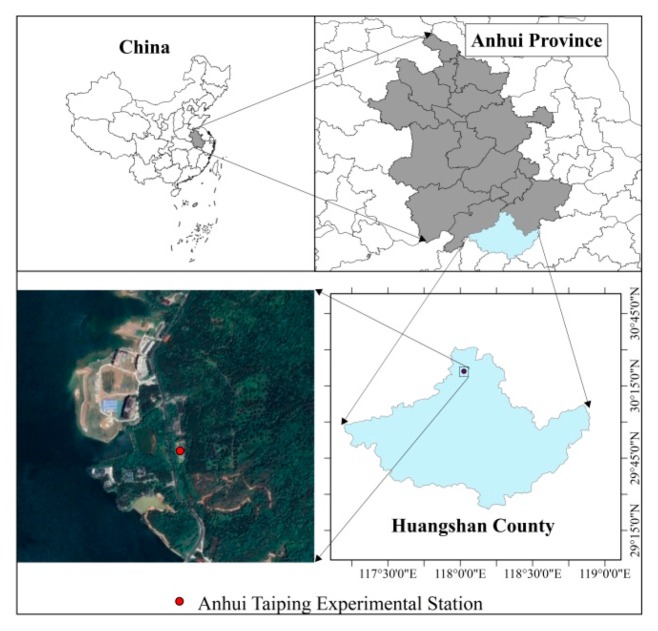
Schematic diagram of the location of *P. edulis* sprouting seedling (test materials) cultivation (30°21′N, 118°01′E).

*P. edulis* is a gramineae, perennial grass. *P. edulis* sprouting seedlings used in this experiment were obtained by seed sowing from seed collected from one mother line, and cultivation in 2015 under the same environmental conditions. They were cultivated in a greenhouse located in the International Center for Bamboo and Rattan’s Anhui Taiping Experimental Station, located in Huangshan, Anhui, China (Figure 8). 

This experimental area experiences a humid subtropical monsoon climate. The average temperature in July is around 25 °C, and the annual average temperature is 15–16 °C. Three-year-old *P. edulis* sprouting seedlings were selected from the greenhouse nursery. Clonal ramets of *P. edulis* consisting of two ramets connected by a rhizome were selected. Selected clonal ramets were all at the same development stage. To obtain similar clones, we selected plants with similar rhizome length and plant growth, consisting of integrated ramets of the same strain. The roots of each clonal branch thus had the same ability to absorb water and nutrients, therefore ensuring their functionally autonomous ability. 

### 3.2. Experiment Design

**Figure 9:**
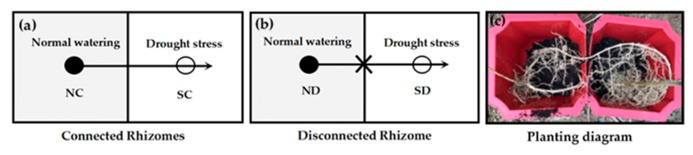
Schematic representation of the experimental design. (**a,b**) The line with the arrow represents the rhizome, the arrow represents the rhizome apex. Cloned ramets grown under normal watering (RWC: 80 ± 5%) and drought stress (RWC: 25 ± 5%) are represented by filled and open circles respectively. (**c**) Clonal strains of *P. edulis* consist of two clonal ramets that were growing in soil with different soil RWC.

Three-year-old sprouted seedlings with similar rhizome characteristics as described in *2.1. Materials* were selected. In the experiment, a two-factor design was used, with two levels of soil relative water content (RWC = 25 ± 5%, 80 ± 5%) crossed with two levels of rhizome treatment (connected or disconnected), resulting in a total of four different treatments (Figure 9). The *P. edulis* sprouting seedlings were henceforth denoted as connected rhizome treatment (NC, SC) and disconnected rhizome treatment (ND, SD) in experiments, where C denoted connected rhizome (with integration), D represented disconnected, severed rhizome (no integration), and N and S were short for normal water treatment or stress (drought) treatment respectively. Each treatment was treated in six replicates. The seedlings to be tested were placed in a plastic box (size 50 cm × 25 cm × 30 cm, long × wide × high). In order to prevent water from seeping between the two ramets, a hole was drilled in the wall of the plastic box for the rhizome to be placed (Figure 9c). After three weeks for recovery (on 10 October 2018), 30 clonal fragments of *P. edulis* were selected and used in the experiment as described below.

Water was added to the *P. edulis* sprouting seedlings test material to saturate the soil. ND and NC treatments were continuously watered to maintain RWC = 80 ± 5%, and the SD and SC parts were subjected to natural transpiration treatment (not adding water), and in line with the drought stress threshold of *P. edulis* sprouting seedlings (RWC was about 30% measured by our research group). All test treatments were sampled and tested until natural transpiration consumption reached a relative water content of 25 ± 5%. RWC was monitored by a soil temperature and humidity meter EM-50 (METER Group, Inc., Pullman, Washington, DC, USA). 

### 3.3. Methods

#### 3.3.1. Laser Confocal Microscopy Luminescence Imaging to Observe the Structural Characteristics of Vascular Bundles of the Stem and Rhizome

Using the characteristic high lignin concentration in the *P. edulis* vascular bundles, the stem and rhizome were observed. Lignin concentration of vascular bundles is directly proportional to the fluorescence intensity of the laser, the structural characteristics of the vascular bundles in the rhizome and stem were observed using Laser confocal microscopy (LSM510, LeicaDM4, Berlin, Germany) at a 488 nm excitation wavelength.

#### 3.3.2. Measurement of Ca^2+^, NH^4+^, and NO^3−^ Flux 

Net Ca^2+^, NH^4+^, and NO^3−^ flux was measured using Non-Invasive Micro-Test Technology (NMT Physiolyzer^®^, Younger USA LLC, Amherst, MA 01002, USA; Xuyue (Beijing) Sci.&Tech. Co., Ltd., Beijing, China) at the Eco-instrument Analysis Lab, College of Biology and the Environment, Nanjing Forestry University (Nanjing, Jiangsu 210037, China). Non-invasive Micro-Test Technology (NMT) can non-invasively measure Ca^2+^, NH^4+^, and NO^3−^ flux with a high level of both temporal and spatial resolution. It measures the concentration gradient of Ca^2+^, NH^4+^, and NO^3−^ by means of a flux microsensor “vibrating” repeatedly between two points in the sample surface. Measurement of Ca^2+^, NH^4+^, and NO^3−^ flux in the test was reported by voltage output [45].

After different test treatments, living samples were incubated in the measuring solution to equilibrate for 10 min (Pileorhiza), 3 h (Rhizome), and 30 min (Mesophyll). Then, samples were transferred to a measuring chamber containing 10–15 mL of a fresh measuring solution. Measuring solution makeup and position site are shown Table 2.

The system setup parameters in the experiment are as follows. Microsensors to measure Ca^2+^, NH^4+^, and NO^3−^ flux (Φ 4.5 ± 0.5 μm) were prepared by backfilling with electrolyte solution (100 mM CaCl_2_, 100 mM NH_4_Cl, and 10 mM KNO_3_) up to approximately 1.0 cm from the tip. The micropipettes were front filled with 40–50 μm columns of selective liquid ion-exchange cocktails (Ca^2+^ LIX, XY-SJ-Ca, Xuyue (Beijing) Sci.&Tech. Co., Ltd., Beijing, China). An Ag/AgCl wire flux microsensor holder YG003-Y11 (Xuyue (Beijing) Sci.&Tech. Co., Ltd., Beijing, China) was inserted in the back of each flux microsensor to make electrical contact with the electrolyte solution. A flux microsensor with a Nernstian slope of >22 mV per decade was used in this study. The same flux microsensor was calibrated again according to the same procedure and standards after each test.

The Ca^2+^, NH^4+^, and NO^3−^ fluxes were calculated by Fick’s law of diffusion as follows:

J = −D·(dc/dx),

where dx (30 μm) is the distance the flux microsensor moved repeatedly from one point to another point perpendicular to the surfaces of samples at a frequency of ca. 0.3 Hz. 

#### 3.3.3. Changes of Hormone, Malondialdehyde (MDA), and Osmotic Adjustment Substance Content of *P. edulis* under Heterogeneous Drought Stress 

The leaves of the clones [NC/ND] and clones [SC/SD] were separately determined by enzyme- linked immunosorbent assay (ELISA, Shanghai Enzyme-linked Biotechnology Co., Ltd., Shanghai, China). MDA, JA, ABA, Auxins (IAA), proline (Pro), and betaine (Bet) concentration was determined [46,47,48]. 

#### 3.3.4. The Capacity of Antioxidant Defense Systems Activity of *P. edulis* under Heterogeneous Drought Stress

Leaves from the clonal ramets NC, ND, SC, and SD were collected, and Superoxide (SOD), peroxidase (POD), and catalase (CAT) activity in each sample was determined using the nitroblue tetrazolium (NBT) photochemical reduction, guaiacol, and theredox methods respectively [49,50].

#### 3.3.5. Statistical Analysis

A two-factor repeated measures statistical analysis of variance was performed using SPSS 18.0 (SPSS Inc., Chicago, IL, USA) to calculate the studentized residual for four separate RWC values (25 ± 5%, 80 ± 5%) and rhizome status (connected or disconnected), and to evaluate whether there were any abnormal values. Principal component analysis (PCA) maps were drawn using R software’s ggplot2 package.

After carrying out the Shapiro–Wilk test, physiological indexes such as leaf hormone concentration, osmotic adjustment substance concentration and enzyme activity were found to approximate a normal distribution (*P* > 0.05). The results showed significant interactive effects between soil water content and rhizome status. The simple effect analysis (LSD, *P* < 0.05) of interactive effects between the connected disconnected rhizome groups was tested using syntax.

Significant differences are denoted with letters of the alphabet. At the same time, SPSS 18.0 was used to analyze the correlation between concentration of JA and ABA, concentration of Pro and MDA, and concentration of Bet and MDA. 

## 4. Conclusions

This study shows that rhizome severance treatment on cloned ramets of *P. edulis* had significant effects on the water stress response. Under heterogeneous water stress, the rhizome that links cloned ramets of *P. edulis* plays an important role in regulating water physiology. LCSM was used to observe the structural characteristics of the stem and rhizome transverse section of *P. edulis*. It was found that the vascular bundle structures were similar in both plants. This is taken to mean that physiological integration provides a structural support of the clonal plant *P. edulis* in a heterogeneous water environment. We also used NMT to detect the Ca^2+^ flux in various *P. edulis* organs under heterogeneous water stress and found that the Ca^2+^ absorption ability in the connected rhizome was strong, and that the difference in flux between the two clonal ramets was small. 

Combined with the related physiological and biochemical indices, this study found that the *P. edulis* rhizome plays an important part in the adaptation of the plant to drought stress, especially under heterogeneous water conditions where the sharing of water through the rhizome is possible. This facilitates the adaptability of the whole plant to drought stress conditions. Most physiological indexes were significantly lower in the SC treatment than in the SD treatment under the same soil moisture content. 

Our findings reveal that the physiological changes caused by the cascade effect of the rhizome tends to be beneficial to the stress response. Compared with the control group with the disconnected rhizome, relevant physiological and biochemical indexes of the cloned ramets with a connected rhizome shifted towards adaptation to the stress. Under heterogeneous drought stress conditions, the benefits of the attached rhizome of clonal *P. edulis* ramets are obvious.

## Figures and Tables

**Table 1:** Analysis of the variance in various physiological indexes of *P. edulis* sprouting seedlings, showing the correlation between these indexes and of rhizome status (R), water stress (W) and a combination of the two (W × R) on *P. edulis*.

	W	R	W × R
SOD activity	2947.21 ***	4.11 ^ns^	979.94 ***
CAT activity	322.15 ***	77.69 ***	155.23 ***
POD activity	137.26 ***	6.54 ^ns^	26.91 ***
JA concentration	999.70 ***	40.41 ***	105.17 ***
ABA concentration	1076.62 ***	303.77 ***	1040.69 ***
IAA concentration	553.84 ***	73.67 ***	243.18 ***
Pro concentration	56.84 ***	0.02 ^ns^	12.87 **
Bet concentration	1860.51 ***	605.20 ***	1898.34 ***
MDA concentration	518.35 ***	8.93 **	430.09 ***

Note: Values are F and symbols show *p* values (*** *p* < 0.001, ** *p* < 0.01, * *p* < 0.05, and ns *p* ≥ 0.05).

**Table 2:** Non-invasive Micro-Test Technology (NMT) test site and test solution constitution.

Ion Species Forflux Testing	Test Position Site (μm)			Measuring Solutions Constitution	
	Pileorhiza	Rhizome	Mesophyll	Concentration	Constitution
Ca^2+^	0 μm from the root apex	Cross-section	Cross-section	0.1 mM 0.1 mM	NH_4_NO_3_ CaCl_2_
NH^4+^	-	-	Cross-section	0.1 mM 0.1 mM	NH_4_NO_3_ CaCl_2_
NO^3−^	-	-	Cross-section	0.1 mM 0.1 mM	NH_4_NO_3_ CaCl_2_
